# Metabolic and Bariatric Surgery Utilization Trends in the United States: Evidence From 2012 to 2021 National Electronic Medical Records Network

**DOI:** 10.1097/AS9.0000000000000317

**Published:** 2023-12-04

**Authors:** Abdulrahman Alsuhibani, Jonathan R. Thompson, Patricia R. Wigle, Jeff Jianfei Guo, Alex C. Lin, Marepalli B. Rao, Ana L. Hincapie

**Affiliations:** From the * Department of Pharmacy Practice, Unaizah College of Pharmacy, Qassim University, Saudi Arabia; † Department of Health outcome, James L. Winkle College of Pharmacy, University of Cincinnati Academic Health Center, Cincinnati, OH; ‡ Department of Surgery, University of Cincinnati College of Medicine, Cincinnati, OH; § Department of Environmental and Public Health Sciences, University of Cincinnati College of Medicine, Cincinnati, OH.

**Keywords:** bariatric surgery procedure trends, metabolic and bariatric surgery, obesity, Roux-en-Y gastric bypass, sleeve gastrectomy, trends, utilization

## Abstract

**Background:**

Bariatric surgery has evolved over the past 2 decades yet assessing trends of bariatric surgery utilization in the growing eligible population is lacking.

**Aim:**

This study aimed to update the trends in bariatric surgery utilization, changes in types of procedures performed, and the characteristics of patients who underwent bariatric surgery in the United States, using real-world data.

**Method:**

This retrospective descriptive observational study was conducted using the TriNetX, a federated electronic medical records network from 2012 to 2021, for adult patients 18 years old or older who had bariatric surgery. Descriptive statistical analysis was conducted to assess patients’ demographics and characteristics. Annual secular trend analyses were conducted for the annual rate of bariatric surgery, and the specific procedural types and proportions of laparoscopic surgeries.

**Results:**

A steady increase in the number of procedures performed in the United States over the first 6 years of the study, a plateau for the following 2 years, and then a decline in 2020 and 2021 (during the coronavirus disease 2019 pandemic). The annual rate of bariatric surgery was lowest in 2012 at 59.2 and highest in 2018 at 79.6 surgeries per 100,000 adults. During the study period, 96.2% to 98.8% of procedures performed annually were conducted laparoscopically as opposed to the open technique. Beginning in 2012, the Roux-en-Y gastric bypass (RYGB) procedure fell to represent only 17.1% of cases in 2018, along with a sharp decline in the adjustable gastric band (AGB) procedure, replaced by a sharp increase in the sleeve gastrectomy (SG) procedure to represent over 74% of cases in 2018.

**Conclusions:**

Bariatric surgery utilization in the United States showed a moderate decline in the number of RYGB procedures, which was offset by a substantial increase in the number of SG procedures and a precipitous drop in the annual number of AGB procedures.

## Abstract 2

Mini-abstract: Bariatric surgery has evolved over the past 2 decades yet assessing trends of bariatric surgery utilization in the growing eligible population is lacking. This study aimed to update the trends in bariatric surgery utilization, changes in types of procedures performed, and the characteristics of patients who underwent bariatric surgery in the United States, using real-world data. This retrospective descriptive observational study was conducted using the TriNetX, a federated electronic medical records network from 2012 to 2021, for adult patients 18 years old or older who had bariatric surgery. Descriptive statistical analysis was conducted to assess patients’ demographics and characteristics. Annual secular trend analyses were conducted for the annual rate of bariatric surgery, and the specific procedural types and proportions of laparoscopic surgeries. A steady increase in the number of procedures performed in the United States over the first 6 years of the study, a plateau for the following 2 years, and then a decline in 2020 and 2021 (during the coronavirus disease 2019 pandemic). The annual rate of bariatric surgery was lowest in 2012 at 59.2 and highest in 2018 at 79.6 surgeries per 100,000 adults. During the study period, 96.2% to 98.8% of procedures performed annually were conducted laparoscopically as opposed to the open technique. Beginning in 2012, the Roux-en-Y gastric bypass (RYGB) procedure fell to represent only 17.1% of cases in 2018, along with a sharp decline in the adjustable gastric band (AGB) procedure, replaced by a sharp increase in the sleeve gastrectomy (SG) procedure to represent over 74% of cases in 2018. Bariatric surgery utilization in the United States showed a moderate decline in the number of RYGB procedures, which was offset by a substantial increase in the number of SG procedures and a precipitous drop in the annual number of AGB procedures.

## INTRODUCTION

Obesity is a chronic disease and medical condition involving an excessive amount of body fat, which can create health problems and complications.^1^ Obesity prevalence in the United States remained relatively stable in the 1960s and 1970s. However, it escalated in the following decades, rising from 30.5% to 42.4% between 1999 and 2018.^2^ According to recent findings, obesity trends among the adult population in the United States reached over 39% and are predicted to reach 51% by 2030 unless it is abated.^3–6^

Bariatric surgery is currently considered the most effective treatment option for morbid obesity and obesity-related comorbidities, as several studies have shown limited efficacy and failure to achieve long-term weight loss with nonsurgical interventions compared with bariatric surgery.^7–9^ However, as the demand for bariatric surgery has increased significantly along with an anticipated increase in the obese population, nationally representative information on changes in the overall bariatric surgery utilization, patient characteristics, types of procedures performed, and the projected trends in the continually growing eligible population is needed for healthcare resource planning and population health.^10–12^

In the 1950s, at the University of Minnesota, the first procedure expressly aimed at weight loss was conducted.^13,14^ However, secular trends in utilizing bariatric surgery in the United States have not been explored before 2002. There are 8 population-based studies that have evaluated bariatric surgery utilization in the United States. Seven studies used the Nationwide Inpatient Sample database, and 1 utilized claims data. Studies that used Nationwide Inpatient Sample data covered the period between 1990 and 2016. The studies have reported a sharp increase in bariatric surgery utilization in the 1990s and a plateau or gradual increase trend after 2004.^15–19^ Moreover, in 2005, laparoscopic techniques surpassed the open bariatric surgery techniques as the most frequently used.^16,19^ Finally, findings from the study by Alalwan et al^18^ that used 2006–2015 claims data revealed that bariatric surgery utilization peaked in 2010 and then plateaued.

A series of surveys sent to the members of the International Federation for Obesity Surgery and Metabolic Diseases in 2003, 2008, 2011, 2013, 2014, and 2016 have been used to estimate the number and type of bariatric surgeries performed worldwide.^20–25^ Unlike multiple studies that used US data and found a plateau trend, these studies found a steady increase in the number of bariatric surgeries conducted during the survey’s iterations. However, since only 35% of national societies had a national registry and most of the data were self-reported, the accuracy of the information elicited from these surveys was a major point of weakness.

Consequently, very few recent studies have evaluated the utilization of bariatric surgery despite the steep increase in obesity prevalence. As such, this study aims to estimate the annual trends of bariatric surgery utilization, changes in the types of procedures performed, and the characteristics of patients who underwent bariatric surgery in the United States from 2012 to 2021 using a national database of aggregated electronic medical records (EMRs) data.

## METHODS

### Study Design and Population

This retrospective descriptive observational study used EMR data for adult patients who had bariatric surgery procedures. To be included in the study, patients had to be 18 years old or older at the time of surgery. This study included laparoscopic and open primary bariatric surgery procedures performed during the study period. Current Procedure Terminology (CPT-4) codes; International Classification of Diseases, Ninth Revision, Clinical Modification (ICD-9-CM); and International Classification of Diseases, Tenth Revision, Clinical Modification (ICD-10-CM) codes were used to identify patients who have had bariatric surgery (Appendix 1, http://links.lww.com/AOSO/A230).^26,27^ All revisional bariatric procedures were excluded (Appendix 2, http://links.lww.com/AOSO/A230).^26,27^

### Data Source

The data was obtained and extracted from the TriNetX (Cambridge, MA) database from January 2012 to December 2021. TriNetX is a Health Insurance Portability and Accountability Act of 1996 compliant health research data aggregator. It is a federated health research network platform with over 84 million patients’ EMR from 69 health care organizations (HCOs) in the United States, comprising primary care, hospitals, and specialty treatment providers. Most of the HCOs are large academic medical centers with inpatient and outpatient services. The TriNetX platform provides access to de-identified clinical data aggregated from participating or member HCOs. Both the patients and the HCOs who provide data remain anonymous.^28–30^

Because only de-identified aggregated patient data were used, our study was exempt from requiring human subject approval from the Institutional Review Board at the University of Cincinnati.

### Exposure

Our definition of exposure is “receiving metabolic or bariatric surgery.” The CPT-4, ICD-9-CM, and ICD-10-CM codes were used to determine the type of procedure received and the service date. Cases were identified either with the presence of at least one of the following CPT-4 codes in outpatient or inpatient records: open Roux-en-Y gastric bypass (RYGB), open sleeve gastrectomy (SG), open biliopancreatic diversion with duodenal switch (BPD/DS) or BPD with gastric reduction duodenal switch, open vertical-banded gastroplasty (VBG), laparoscopic RYGB, laparoscopic SG, laparoscopic adjustable gastric band (AGB), laparoscopic single anastomosis duodeno-ileal bypass with sleeve gastrectomy (SADI-S), or with an ICD-9-CM/ICD-10-CM obesity codes plus one of the following codes for bariatric procedures in outpatient or inpatient records: “open RYGB, open SG, open BPD/DS or BPD with gastric reduction duodenal switch, open VBG, laparoscopic RYGB, laparoscopic SG, laparoscopic AGB.” (Appendix 1, http://links.lww.com/AOSO/A230).

### Statistical Analyses

Baseline characteristics and demographics were reported as means ± standard deviations for continuous variables, and as frequencies (N) and percentages (%) for categorical variables. The annual rates of bariatric surgery per 100,000 adults for each year were calculated using the total number of bariatric surgical procedures obtained from the TriNetX database as a numerator and the number of adult individuals (age ≥ 18 years) with at least 1 hospital visit as the denominator for each year. TriNetX allows real-time data access, enabling queries using standardized terminologies such as CPT and ICD codes. Therefore, the statistical tools within the platform were queried to generate results for this study. Microsoft Excel software (Microsoft Corporation, Redmond, WA) have been used to create all figures and graphs.

## RESULTS

### Patient Characteristics

We identified 206,953 adults who had undergone bariatric surgery between January 1, 2012, and December 31, 2021. The mean age decreased from 51.7 years (standard deviation of the mean, 11.9 years) in 2012 to 41.8 years (standard deviation of the mean, 11.9 years) in 2021, with the proportion of female ranging between 79.3% and 84.1% (Table 1). The proportion of white patients also decreased across years from 75% in (2012) to 63.4% in (2021), while Black/African American and Hispanic/Latino patients have increased over the years. From 2012 to 2021, there was an 9.1 increase in the percentage of Black patients and 5.5% increase in Hispanic patients who had received the surgery. The South and Northeast regions of the United States had the highest rates of bariatric surgery across the study period, ranging from 37.2% to 44.5% and 35.6% to 40.4%, respectively.

**TABLE 1.** Demographics and Characteristics of the Patient Population Who Underwent Bariatric Surgery in the United States, 2012–2021

Variables	2012	2013	2014	2015	2016	2017	2018	2019	2020	2021
Total number of bariatric surgeries	14,794	15,994	18,392	18,184	19,764	23,672	24,323	25,053	22,109	24,668
Age										
Years, mean (SDM)	51.6 (11.6)	51.7 (11.9)	51.3 (12.3)	49.2 (12.2)	48.7 (12.3)	47.2 (12.2)	46.1 (12.1)	45.2 (12.1)	43.5 (12.2)	41.8 (11.9)
Sex, N (%)										
Male	2914 (19.7)	3311 (20.7)	3660 (19.9)	3728 (20.5)	3873 (19.6)	4521 (19.1)	4719 (19.4)	4660 (18.6)	3847 (17.4)	3922 (15.9)
Female	11,880 (80.3)	12,683 (79.3)	13,732 (80.1)	14,456 (79.5)	15,891 (80.4)	19,151 (80.9)	19,604 (80.6)	20,393 (81.4)	18,262 (82.6)	20,746 (84.1)
Race, N (%)										
White	11,096 (75.0)	11,900 (74.4)	12,838 (69.8)	12,311 (67.7)	13,677 (69.2)	16,452 (69.5)	16,929 (69.6)	16,936 (67.6)	14,106 (63.8)	15,640 (63.4)
Black or African American	2426 (16.4)	2927 (18.3)	3770 (20.5)	3800 (20.9)	4585 (23.2)	5539 (23.4)	5497 (22.6)	5762 (23.0)	5660 (25.6)	6290 (25.5)
Asian	15 (0.1)	32 (0.2)	55 (0.3)	73 (0.4)	99 (0.5)	96 (0.4)	98 (0.4)	102 (0.4)	134 (0.6)	103 (0.4)
American Indian or Alaska	44 (0.3)	48 (0.3)	74 (0.4)	92 (0.5)	59 (0.3)	70 (0.3)	72 (0.3)	98 (0.4)	65 (0.3)	95 (0.4)
Native Hawaiian or other	15 (0.1)	15 (0.1)	18 (0.1)	17 (0.1)	40 (0.2)	24 (0.1)	24 (0.1)	25 (0.1)	22 (0.1)	24 (0.1)
Unknown	1198 (8.1)	1072 (6.7)	1637 (8.9)	1891 (10.4)	1304 (6.6)	1491 (6.3)	1703 (7.0)	2130 (8.5)	2122 (9.6)	2516 (10.2)
Ethnicity, N (%)										
Not Hispanic or Latino	12,028 (81.3)	12,635 (79.0)	14,162 (77.0)	13,638 (75.0)	15,574 (78.8)	18,819 (79.5)	18,778 (77.2)	19,291 (77.0)	17,311 (78.3)	19,537 (79.2)
Hispanic or Latino	991 (6.7)	912 (5.7)	1177 (6.4)	1127 (6.2)	1087 (5.5)	1965 (8.3)	2578 (10.6)	2405 (9.6)	2366 (10.7)	3010 (12.2)
Unknown	1775 (12.0)	2447 (15.3)	3053 (16.6)	3419 (18.8)	3103 (15.7)	2888 (12.2)	2967 (12.2)	3357 (13.4)	2432 (11.0)	2121 (8.6)
Marital status, N (%)										
Married	2885 (19.5)	3935 (24.6)	4157 (22.6)	4419 (24.3)	4961 (25.1)	5705 (24.1)	5813 (23.9)	6464 (25.8)	5350 (24.2)	6217 (25.2)
Single	2115 (14.3)	3103 (19.4)	3660 (19.9)	3328 (18.3)	3874 (19.6)	4285 (18.1)	4986 (20.5)	5437 (21.7)	4732 (21.4)	5402 (21.9)
Unknown	9794 (66.2)	8956 (56)	10,575 (57.5)	10,437 (57.4)	10,929 (55.3)	13,682 (57.8)	13,524 (55.6)	13,152 (52.5)	12,027 (54.4)	13,049 (52.9)
Regions, N (%)										
South	5696 (38.5)	6813 (42.6)	7283 (39.6)	6892 (37.9)	7352 (37.2)	10,416 (44.0)	9851 (40.5)	10,322 (41.2)	9109 (41.2)	10,977 (44.5)
Northeast	5267 (35.6)	5822 (36.4)	6952 (37.8)	6601 (36.3)	7807 (39.5)	8593 (36.3)	10,167 (41.8)	10,372 (41.4)	9242 (41.8)	9966 (40.4)
Midwest	2559 (17.3)	2239 (14.0)	3108 (16.9)	3091 (17.0)	3261 (16.5)	2651 (11.2)	2675 (11.0)	2806 (11.2)	2697 (12.2)	2713 (11.0)
West	1272 (8.6)	1120 (7.0)	1048 (5.7)	1600 (8.8)	1324 (6.7)	1894 (8.0)	1362 (5.6)	1052 (4.2)	1017 (4.6)	987 (4.0)
Unknown	0.0	0.0	0.0	0.0	20 (0.1)	118 (0.5)	268 (1.1)	501 (2.0)	44 (0.2)	25 (0.1)

SDM indicates standard deviation of the mean.

### Volume and Rates of Bariatric Surgery

**FIGURE 1.**
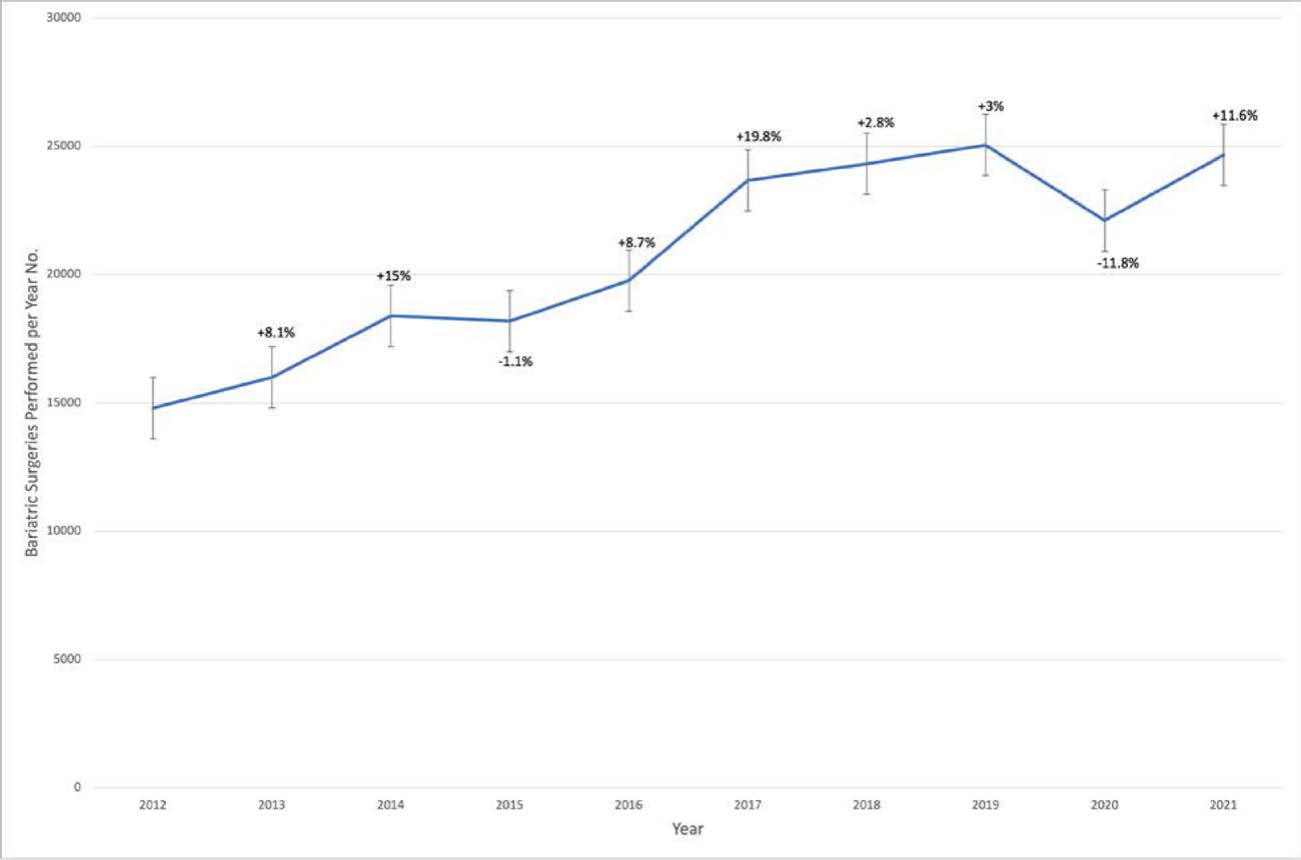
Number of metabolic and bariatric surgery utilization and percent change from previous year in the United States, 2012–2021.

During the study period, bariatric surgery volume was the lowest in 2012 at 14,794 procedures and the highest in 2019 at 25,053 procedures (Table 2). Figure 1 depicts the year-to-year percent change in bariatric surgery utilization over the study period, where the highest increase was in 2017, with an over 19% increase, while the largest decline occurred in 2020 (–12%).

**Table 2.** Utilization Changes by Bariatric Surgery and Surgery Technique Types Performed, 2012–2021

Variables	2012	2013	2014	2015	2016	2017	2018	2019	2020	2021
Surgeries No.	14,794	15,994	18,392	18,184	19,764	23,672	24,323	25,053	22,109	24,668
Type of surgery technique used No. (%)										
Lap	14,227 (96.2)	15,471 (96.7)	17,855 (97.1)	17,783 (97.8)	19,385 (98.1)	23,240 (98.2)	23,902 (98.3)	24,620 (98.3)	21,852 (98.8)	24,332 (98.6)
Open	567 (3.8)	523 (3.3)	537 (2.9)	401 (2.2)	379 (1.9)	432 (1.8)	421 (1.7)	433 (1.7)	257 (1.2)	336 (1.4)
Type of bariatric procedures performed No. (%)										
RYGB	5,733 (38.8)	5,363 (33.5)	5,167 (28.1)	4,636 (25.5)	4,559 (23.1)	4,723 (20.0)	4,166 (17.1)	4,367 (17.4)	4,033 (18.2)	4,738 (19.2)
SG	5,234 (35.4)	7,481 (46.8)	10,428 (56.7)	11,349 (62.4)	13,490 (68.3)	16,944 (71.6)	18,213 (74.9)	18,838 (75.2)	15,934 (72.1)	17,938 (72.7)
AGB	2,648 (17.9)	1,792 (11.2)	1,532 (8.3)	855 (4.7)	622 (3.1)	575 (2.4)	409 (1.7)	338 (1.3)	357 (1.6)	305 (1.2)
BPD/DS	184 (1.2)	177 (1.1)	161 (0.9)	134 (0.7)	120 (0.6)	282 (1.2)	265 (1.1)	221 (0.9)	308 (1.4)	268 (1.1)
VBG	32 (0.2)	17 (0.1)	26 (0.1)	46 (0.3)	114 (0.6)	108 (0.5)	103 (0.4)	107 (0.4)	89 (0.4)	81 (0.3)
SADI_S	963 (6.5)	1,164 (7.3)	1,078 (5.9)	1,164 (6.4)	859 (4.3)	1,040 (4.4)	1,167 (4.8)	1,182 (4.7)	1,388 (6.3)	1,338 (5.4)

AGB indicates adjustable gastric band; BPD/DS, biliopancreatic diversion with duodenal switch; RYGB, Roux-en-Y gastric bypass; SADI-S, single anastomosis Duodenal-Ileal bypass with sleeve gastrectomy; SG, sleeve gastrectomy; VBG, vertical-banded gastroplasty.

**FIGURE 2.**
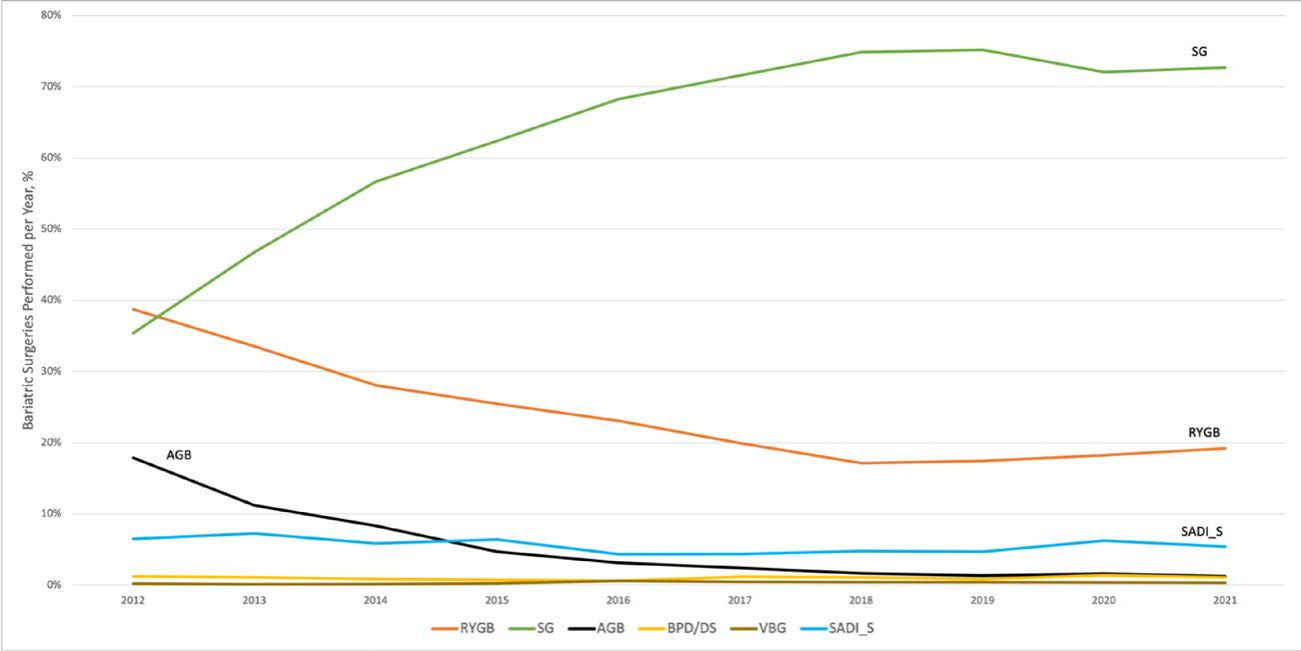
Annual change in the proportion of bariatric surgery procedures performed in the United States, 2012–2021.

SG and RYGB procedures accounted for the majority of all performed procedures (ranging from 35.4% to 75.2% and 17.1% to 38.8% of the annually performed procedures, respectively). In 2012, the proportion of both SG and RYGB had comparable rates of 35.5% and 38.8%, respectively. However, the proportion of RYGB decreased to 17.1% in 2018, while the proportion of SG increased to 75.2% in 2019 (Table 2). The highest proportion for the AGB was in 2012 at 17.9%, after which it decreased throughout the study period to reach its lowest proportion in 2021 (1.2%). As shown in Figure 2, the most frequently utilized procedure has been SG, followed by RYGB, while SADI-S, VBG, and BPD/DS usage has remained consistent throughout the study period, with a rare utilization of the VBG and BPD/DS procedures. As displayed in Table 2, laparoscopic techniques were most frequently performed and their use rate continued to rise, reaching an all-time high in 2020 (98.8%).

**FIGURE 3.**
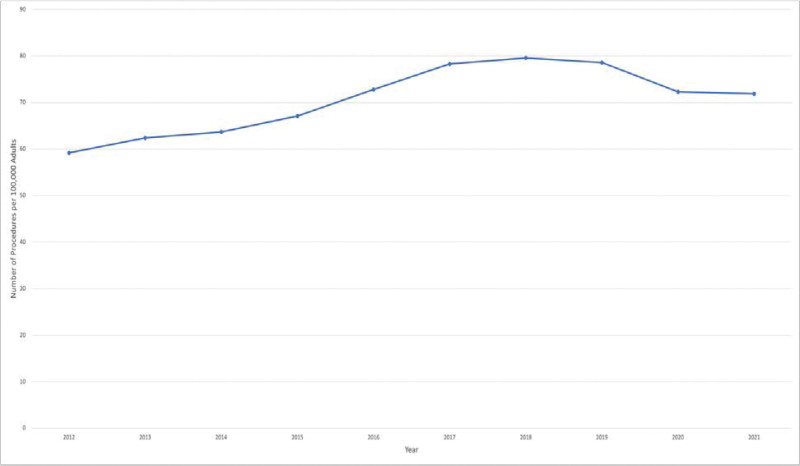
Metabolic and bariatric surgery utilization annual rate per 100,000 adults in the United States, 2012–2021.

In the first 6 years of the study period, the annual rate of bariatric surgery steadily increased. It remained constant in the 2 years that followed before a decline in 2020 and 2021 (Fig. 3). The rate gradually increased from 59.2 procedures per 100,000 adults in 2012 to 78.3 procedures per 100,000 adults in 2017. The annual rate increased to 79.6 procedures in 2018, peaked that year, before falling to 72.3 surgeries per 100,000 adults in 2020.

## DISCUSSION

Using the TriNetX EMRs database to analyze the utilization trends of bariatric surgery, we observed a steady rise in the number of procedures carried out in the United States over the first 6 years of the study, a plateau for the following 2 years, and then a decline in 2020 and 2021 during the coronavirus disease 2019 (COVID-19) pandemic, as noted in a previous publication.^31^ This might be due to COVID-19 leading to some hospitals halting elective procedures and patients concerns since obesity is such a big risk factor for poor COVID-19 outcomes.^31,32^ According to this study, the population-based annual rate of bariatric surgery was lowest in 2012 at 59.2 surgeries per 100,000 adults and highest in 2018 at 79.6 surgeries per 100,000 adults. Between 96.2% and 98.8% of procedures were conducted laparoscopically as opposed to the open technique, which may be explained by the short hospital stay, surgery time, and precision of the laparoscopic technique. However, bariatric surgery has seen a significant transformation. Beginning in 2012, the RYGB procedure fell to represent only 17.1% of cases in 2018, along with a sharp decline in the AGB procedure. In contrast, the SG procedure sharply rose to over 74% of cases in 2018. The ongoing growth of evidence from the systematic reviews and meta-analyses of all relevant randomized controlled trials supports that; better access to care, outreach from specialized societies, and media platforms communications are some of the suggested variables that could have contributed to this rise.^33–35^

The observed 10-year decrease in the average age of patients undergoing bariatric surgery between 2012 and 2021 is a noteworthy finding, potentially reflecting a shift in patients seeking bariatric interventions at a younger age. Various factors may contribute to this trend, including heightened awareness of obesity-related health risks, improved access to information, and advances in surgical techniques, making the procedure safer and more acceptable to younger patients.^36,37^ Further research is needed to elucidate the precise reasons behind this demographic shift and its implications for the long-term outcomes of bariatric surgery.

The percentages of the different bariatric procedure types conducted to the total number of procedures have undergone significant changes. The results reveal that SG overtook RYGB in 2013, and this growth persisted throughout the study period as SG was more prevalent in the United States. However, up until 2013, RYGB was still the most common bariatric treatment globally.^21^ The steep increase in performing the SG compared with the RYGB procedure can be attributed to the simplicity nature of this surgical technique, in addition to the long-term proof of the favorable safety and efficacy outcomes.^38–40^ The results of this study also support the sharp decline in AGB utilization that has been observed globally, which may be related to the relatively modest degree of weight loss compared with other procedures.^41^ Along with BPD/DS and VBG, the utilization of SADI-S bariatric procedures has remained stable throughout the study period.

The current findings of this study confirm earlier findings that bariatric surgery is still underutilized, despite the growing trends of the eligible obese population in the United States and worldwide, the improvements in preoperative safety, and the well-documented benefits of bariatric surgery. According to previous estimations, when only primary procedures are considered, approximately 1.1% of all patients eligible for bariatric surgery choose surgery as a form of treatment.^35,42^ Understanding the reasons behind the relatively low utilization is needed to support studying and overcoming the barriers to access to surgical care. The American Society for Metabolic and Bariatric Surgery and the International Federation for Obesity Surgery and Metabolic Diseases have recently updated the candidacy criteria for metabolic and bariatric surgery, aiming to increase obese patients’ accessibility to perform the procedure and benefit from its potential advantages of enhancing quality of life, cardiometabolic and mental health, and life expectancy.^19,43^ However, it is necessary to acknowledge that there is a need to factor in all treatment agents that might have an impact on weight loss and whether it affects the surgery utilization.

Even though we saw an increase in the number of Black or African American and Hispanic patients who underwent bariatric surgery, racial disparity in bariatric surgery utilization still persists, as previous publications have suggested that minorities might not have the same access to the procedure as White people.^19,44^ The present study’s results indicate around a 12% decrease in bariatric surgery utilization over the study period among Whites, a 9% increase among those of Black or African descent, and no appreciable changes among other ethnic groups. The current best estimates indicate that among 40- to 59-year-old women, roughly 52% of non-Hispanic Black individuals and 47% of Hispanic individuals are obese compared with 36% of non-Hispanic White individuals.^45^ Given the higher prevalence of obesity and metabolic complications among minority groups, it is possible that minorities do not receive bariatric surgery as frequently as they should.^19,46^

This study investigated the annual rate of bariatric surgery utilization using recent multiyear EMRs data obtained from the TriNetX network that is not confined to patients receiving health coverage from a private or public insurer. The length of the study period, the large and geographically diverse US bariatric surgery patient population, and the use of recent data allowed a thorough examination of the current trends of bariatric surgery. Along with strengths, there are a few limitations in this study. First, as with all retrospective studies using routinely collected data, the dependency on the quality and completeness of the data recorded and how it was recorded within the database. In this regard, the TriNetX data are confined to EMRs gathered as per standard clinical practice; no additional chart review data is provided. The TriNetX data were not acquired especially for research purposes. Therefore, there is a chance for miscoding the diagnoses and clinical events. Second, while the EMR databases may offer more current, thorough, and accurate patient health information than claims data, it only captures data from participating healthcare systems inside the research network and does not include information from other doctors or providers. A third limitation is that some demographic variables (eg, body mass index, insurance status, and income) and clinical data were not included in the data. A fourth limitation of this study pertains to the absence of a designated CPT code for the SADI bariatric surgery procedure. Consequently, our analyses relied on the utilization of an unlisted CPT code (43999), which may introduce potential inconsistencies in reporting and data accuracy. Finally, all revisional procedures were excluded, as stated in the “Methods” section, but some patients who had previously undergone AGB might have undergone SG or RYGB as a revisional procedure, which was recognized as primary surgery. Likewise, patients who had undergone a prior SG may have had a subsequent resleeve, RYGB, SADI-S, or BPD/DS. Despite these limitations, this study provides clinicians, payers, decision-makers, and other stakeholders with valuable information on future projections and the current trends of bariatric surgery in the United States. These findings represent real-world patterns that may shed light on the use and demand for bariatric surgery as a result of the rising obesity rates.

## CONCLUSIONS

In conclusion, the annual rates of bariatric surgery in the United States show a gradual increase between 2012 and 2017, a plateau in 2018 and 2019, before it declined in 2020 and 2021. The percentage of surgeries performed laparoscopically remains high at 96.2% to 98.8% of the cases. The major findings from this study were the moderate decline in the number of RYGB cases, which was offset by a substantial increase in the number of SG cases, and a precipitous drop in the number of AGB procedures performed annually.

## ACKNOWLEDGMENTS

The contributions of the authors to this article are as follows: A.A., J.R.T., P.R.W., J.J.G., M.B.R., and A.L.H. The conceptualization of the study was done by A.A., J.R.T., P.R.W., and A.L.H. Methodology was developed by the same authors along with J.J.G. A.A. conducted data collection and analysis with assistance from M.B.R. and A.L.H. Writing—original draft was completed by A.A. All authors reviewed and edited the article, with contributions from P.R.W., J.J.G., A.C.L., M.B.R., and A.L.H. A.L.H. supervised the project. All authors have given final approval of the version submitted for publication.

## Supplementary Material


