# Supplementary material for: Metabolic and Bariatric Surgery Utilization Trends in the United States: Evidence From 2012 to 2021 National Electronic Medical Records Network
Source: Ann Surg Open. 2023 Dec 4;4(4):e317. doi: 10.1097/AS9.0000000000000317 (PMC10735086; doi:10.1097/AS9.0000000000000317)
Supplement: Supplementary file 1 [file as9-4-e317-s001.pdf]

Metabolic and Bariatric Surgery Utilization Trends in the United States: Evidence from 2012–2021 National Electronic Medical Records Network

Appendices

Table 1. Diagnosis and Procedure Codes for Bariatric Surgery and Obesity

Variable	Codes
Open Roux-en-Y gastric bypass (RYGB)	CPT-4: 43846, 43847<br>ICD-9-CM: 44.31, 44.39<br>ICD-10: 0D16078,
Laparoscopic Roux-en-Y gastric bypass (RYGB)	CPT-4: 43644, 43645<br>ICD-9-CM: 44.38<br>ICD-10: 0D16479, 0D1647A, 0D164J9, 0D164JA, 0D164K9, 0D164KA, 0D164Z9,<br>0D164ZA, 0D164ZB
Open Sleeve gastrectomy (SG)	CPT: 43843,<br>ICD-9-CM: 43.89, 44.69<br>ICD-10: 0DQ60ZZ
Laparoscopic Sleeve gastrectomy (SG)	CPT: 43775<br>ICD-9-CM: 43.82<br>ICD-10: 0DB64Z3
Laparoscopic adjustable gastric band (AGB)	CPT-4: 43770, S2082<br>ICD-9: 44.95<br>ICD-10: 0DV64CZ
Laparoscopic single anastomosis duodenal-ileal bypass with sleeve (SADI-S)	CPT-4:* 43999
Biliopancreatic Diversion with Duodenal Switch (BPD/DS) or Gastric Reduction Duodenal Switch (BPD/GRDS)	CPT-4: 43845, (without Duodenal Switch)<br>ICD-9-CM: 45.91, 45.51, 43.89<br>ICD-10: 0D190Z9, 0DB60ZZ, 0DB80ZZ
Open Vertical-banded gastroplasty (VBG)	CPT: 43842 "Vertical-banded gastroplasty, not performed anymore", VGB procedures are essentially no longer performed.<br>ICD-9-CM: 44.68<br>ICD-10: 0DQ64ZZ
Obesity	ICD-9-CM: 278.0x, 278.01, V77.8, V85.41, V85.42, V85.43, V85.44, V85.45,<br>ICD-10: E66.xx, Z68.4x
Morbid obesity	ICD-9: 278.01<br>ICD-10: E66.01

CPT-4, Current Procedure Terminology (American Medical Association, Chicago, IL),
ICD-9-CM, International Classification of Diseases, Ninth Revision, Clinical Modification,
ICD-10-CM, International Classification of Diseases, Tenth Revision, Clinical Modification.
* Currently, there is no specific CPT code that describes the SADI bariatric surgery procedure. However, some surgeons may use an unlisted CPT code such as 43999 (Unlisted procedure, digestive system) to report the procedure.

Table 2. Procedure Codes Used to Identify Open and Laparoscopic Revisional Bariatric Surgery

Description	Code	Code Type
Laparoscopy, surgical, gastric restrictive procedure; removal of adjustable gastric restrictive device component only	43772	CPT-4
Revision of gastrojejunal anastomosis (gastrojejunostomy) with reconstruction, with or without partial gastrectomy or intestine resection; with vagotomy	43865	CPT-4
Revision of gastroduodenal anastomosis (gastroduodenostomy) with reconstruction; without vagotomy	43850	CPT-4
Revision of gastroduodenal anastomosis (gastroduodenostomy) with reconstruction; with vagotomy	43855	CPT-4
Laparoscopy, surgical, gastric restrictive procedure; revision of adjustable gastric restrictive device component only	43771	CPT-4
Laparoscopy, surgical, gastric restrictive procedure; removal and replacement of adjustable gastric restrictive device component only	43773	CPT-4
Revision of gastrojejunal anastomosis (gastrojejunostomy) with reconstruction, with or without partial gastrectomy or intestine resection; without vagotomy	43860	CPT-4
Revision, open, of gastric restrictive procedure for morbid obesity, other than adjustable gastric restrictive device (separate procedure)	43848	CPT-4
Laparoscopy, surgical, gastric restrictive procedure; removal of adjustable gastric restrictive device and subcutaneous port components	43774	CPT-4
Gastric restrictive procedure, open; removal of subcutaneous port component only	43887	CPT-4
Gastric restrictive procedure, open; revision of subcutaneous port component only	43886	CPT-4
Gastric restrictive procedure, open; removal and replacement of subcutaneous port component only	43888	CPT-4
Laparoscopic removal of gastric restrictive device(s)	44.97	ICD-9
Laparoscopic revision of gastric restrictive procedure	44.96	ICD-9
Open revision of RYGB	44.5	ICD-9
